# Can medical therapy mimic the clinical efficacy or physiological effects of bariatric surgery?

**DOI:** 10.1038/ijo.2013.205

**Published:** 2013-12-03

**Authors:** A D Miras, C W le Roux

**Affiliations:** 1 Molecular and Metabolic Imaging Group, Imperial College London, MRC Institute of Clinical Sciences, London, UK; 2 Department of Experimental Pathology, Conway Institute, School of Medicine and Medical Sciences, University College Dublin, Dublin, Ireland

**Keywords:** medical bypass, gut hormones, pharmacotherapy, lifestyle intervention, devices

## Abstract

The number of bariatric surgical procedures performed has increased dramatically. This review discusses the clinical and physiological changes, and in particular, the mechanisms behind weight loss and glycaemic improvements, observed following the gastric bypass, sleeve gastrectomy and gastric banding bariatric procedures. The review then examines how close we are to mimicking the clinical or physiological effects of surgery through less invasive and safer modern interventions that are currently available for clinical use. These include dietary interventions, orlistat, lorcaserin, phentermine/topiramate, glucagon-like peptide-1 receptor agonists, dipeptidyl peptidase-4 inhibitors, pramlintide, dapagliflozin, the duodenal–jejunal bypass liner, gastric pacemakers and gastric balloons. We conclude that, based on the most recent trials, we cannot fully mimic the clinical or physiological effects of surgery; however, we are getting closer. A ‘medical bypass' may not be as far in the future as we previously thought, as the physician's armamentarium against obesity and type 2 diabetes has recently got stronger through the use of specific dietary modifications, novel medical devices and pharmacotherapy. Novel therapeutic targets include not only appetite but also taste/food preferences, energy expenditure, gut microbiota, bile acid signalling, inflammation, preservation of β-cell function and hepatic glucose output, among others. Although there are no magic bullets, an integrated multimodal approach may yield success. Non-surgical interventions that mimic the metabolic benefits of bariatric surgery, with a reduced morbidity and mortality burden, remain tenable alternatives for patients and health-care professionals.

## Introduction

Obesity is an increasingly prevalent worldwide health problem. Approximately one-third of US adults are obese, and obesity rates have increased dramatically in the past 20 years.^1^ The health consequences of obesity are numerous, with attendant increases in the risk of coronary heart disease, type 2 diabetes mellitus (T2DM), hypertension, dyslipidemia, stroke and certain cancers.^2^ Specific causes are still unclear; however, it is likely that a combination of metabolic, genetic, psychological and environmental factors all contribute to the obesity epidemic.

The number of bariatric surgical procedures performed has also increased dramatically. The appropriate use of bariatric surgery remains a subject of debate, with many physicians in the field remaining sceptical about it, in view of the risks associated with surgery. Ultimately, less invasive treatments are needed to address obesity and associated T2DM in a wider population of affected individuals. This review will discuss the clinical and physiological changes observed following bariatric surgery and examine how close we are to mimicking them through less invasive and potentially safer interventions. We have limited our discussion to the most modern non-surgical treatments that are currently available for clinical use in Europe and/or the in United States.

## Methods

The source was a PubMed search used to identify relevant literature to the clinical efficacy and physiological effects of bariatric surgery procedures, lifestyle interventions, modern pharmacotherapy and less invasive devices on both obesity and T2DM. In view of the wide scope of the review, preferably randomised controlled clinical trials (RCTs) and definitive basic and clinical science publications were chosen with a particular focus to those published 2009–2013.

### Types and clinical effectiveness of bariatric surgery

**Figure 1:**
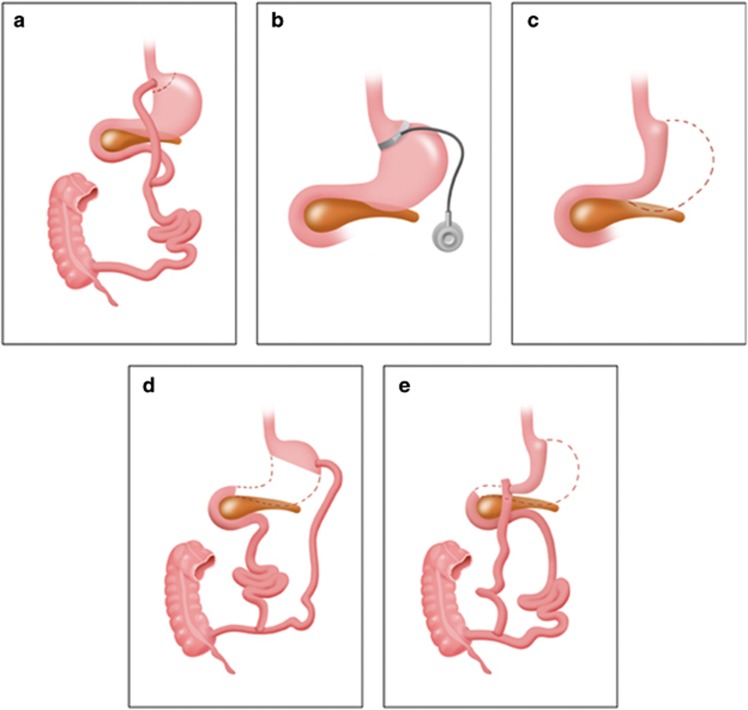
Anatomical manipulation of the surgical bariatric procedures. Bariatric procedures: (**a**) Roux-en-Y gastric bypass; (**b**) adjustable gastric banding; (**c**) vertical sleeve gastrectomy; (**d**) biliopancreatic diversion; (**e**) biliopancreatic diversion with duodenal switch.

Bariatric surgery has been shown to be the most effective treatment for obesity and T2DM, both in large well-matched clinical studies and RCTs.^3, 4, 5, 6, 7, 8^ Roux-en-Y gastric bypass (RYGB) and the adjustable gastric band (AGB) are the most commonly performed surgical procedures around the world. The RYGB procedure typically involves fashioning a 15- to 20-ml gastric pouch and creating a large new outlet that rapidly empties into the mid small intestine (Figure 1). The continuity of the bowel is restored via a jejuno–jejunal anastomosis, between the excluded biliopancreatic limb and the alimentary limb, performed 75–150 cm distally to the gastrojejunostomy.^9^ The gastric remnant is no longer exposed to food; gastric, pancreatic and biliary secretions still flow undiluted in the biliopancreatic limb and come in contact with food in the jejuno–jejunal anastomosis. It is normally performed laparoscopically and causes 25–30% weight loss, which is maintained for at least 20 years.^4, 10^

The AGB technique involves the insertion of an adjustable silicone ring around the proximal aspect of the stomach, immediately below the gastro–oesophageal junction creating a small proximal pouch. The volume of fluid in the band is adjusted through injections in a subcutaneous port. The procedure results in 20–25% long-term weight loss.^10, 11^ The vertical sleeve gastrectomy (VSG) is fashioned through the reduction in gastric volume by the laparoscopic removal of 70–80% of the stomach. Previously, VSG was performed as part of the duodenal switch procedure but is increasingly used as a stand-alone procedure that can cause a weight loss of 20–30% in the long term.^12^ Owing to increased rates of postoperative and nutritional complications, the biliopancreatic diversion and duodenal switch procedures are performed less frequently compared with the other procedures.^10, 11^

Bariatric surgery also results in significant glycaemic improvements in T2DM. Four RCTs have compared RYGB, AGB, VSG and biliopancreatic diversion to lifestyle and pharmacological interventions for obese patients with T2DM.^5, 6, 7, 8^ Their results are consistent in that each of the procedures was superior to non-surgical therapies in terms of reductions in weight, glycaemia and glucose-lowering medication use.

The benefits of bariatric surgery extend beyond improvements in weight and glycaemic control; patients also exhibit reductions in overall and cardiovascular morbidity and mortality rates, as well as a reduction in cancer incidence.^3, 4, 13^ Although some early improvements in diabetic retinopathy and nephropathy have been observed,^14^ the long-term impact of bariatric surgery on microvascular complications is not known. Currently, there are no data from RCTs to support the use of surgery for comorbidities that are frequently associated with obesity including non-alcoholic fatty liver disease, subfertility, renal disease and functional impairment. In terms of obstructive sleep apnoea, AGB resulted in greater weight loss but did not improve the apnoea hypopnoea index significantly more than non-surgical weight loss therapies.^15^ In the absence of sufficient evidence, the choice of technique depends on patient and multidisciplinary team preference, local expertise and funding.

### Complications of bariatric surgery

As with any intervention, bariatric surgery is not without complications. The most serious complications associated with bariatric surgery include postoperative sepsis, anastomotic leaks, bleeding and venous thromboembolism, including fatal pulmonary embolism.^16^ The risk of early mortality after bariatric surgery ranges from 0.1 to 2.0% depending on the procedure.^10^ The longitudinal assessment of bariatric surgery consortium reported a 30-day postoperative mortality rate of 0.3% with RYGB.^17^ Factors associated with increased mortality include male gender, age older than 65 years, reduced cardiorespiratory fitness levels and limited surgeon experience.^16^

Long-term nutritional deficiencies may occur in some bariatric surgery patients due to changes in the anatomy of the gastrointestinal tract with surgery.^18^ Deficiencies in vitamin B_12_, folate and iron are not uncommon early after surgery and evidence of calcium, vitamin D and trace element deficiencies can also occur months to years after the procedure.^18^

Although bariatric surgery has known beneficial effects in terms of T2DM remission, it may result in recurrent postprandial hypoglycaemia in some patients, which may signal an extreme metabolic reaction to surgery. The Swedish bariatric surgery registry found that the risk of hypoglycaemia and related diagnoses were higher in bariatric surgery patients compared with the general population, although the absolute risk of these events remained small.^19^

Overall, modern bariatric surgery has an acceptable risk/benefit profile, with careful patient selection and the availability of an appropriately experienced multidisciplinary team that is responsible for patient care in both the preoperative and postoperative periods.

### Appetite, energy expenditure and food preferences after bariatric surgery

Although RYGB was originally designed to cause gastrointestinal restriction and malabsorption, an increasing volume of evidence supports that the surgery works by reducing hunger and increasing satiation after a meal.^20^ A number of studies suggest that diet-induced energy expenditure increases after RYGB, further aiding weight loss.^21, 22^ Patients also witness a healthy shift in eating behaviour, away from calorically dense high-fat and sweet food to low-calorie alternatives.^23^ The mechanisms behind this are not fully clear yet but may include alterations in food reward, changes in taste function or other consequences relating to the dumping syndrome.^24, 25^ Contrary to malabsorptive procedures, such as the biliopancreatic diversion and duodenal switch, RYGB results in minimal fat and no carbohydrate malabsorption.^26, 27^

There is intense interest in the mechanisms through which VSG and AGB work. Following VSG, patients report reductions in appetite to those experienced after RYGB.^28^ Energy expenditure appears to remain either stable or shows a tendency to decrease in animal models of VSG.^29, 30^ In terms of the AGB, higher intraluminal pressure on vagal afferents in the stomach may facilitate reductions in hunger and meal size after AGB.^31, 32^ Food choices may either not change or even deteriorate,^33^ whereas energy expenditure has not been quantified after AGB.

Gut hormones are some of the mediators of reduced hunger, increased satiation and consequent weight loss after RYGB and VSG surgery. Specifically, RYGB and VSG have been shown to enhance meal-stimulated hormonal secretion of anorexigenic insulin, peptide tyrosine tyrosine (PYY), glucagon-like peptide 1 (GLP-1),^20, 28, 34^ which is sustained for at least 10 years (in the case of RYGB).^35^ The rise in levels of these hormones after VSG is intriguing, but may be because of increased gastric emptying, propelling nutrients to the mid and distal intestine.^36^ The AGB does not significantly alter the plasma levels of these hormones.^20, 37^

Owing to the variability in the laboratory techniques used to measure orexigenic ghrelin, it is currently unclear what happens to this hormone after RYGB;^38^ however, reasonable evidence exists that it decreases after VSG and increases after AGB.^39^ Obestatin, which is a hormone produced from the ghrelin gene but has anorexigenic action and antagonises the effects of ghrelin, has been shown to increase after VSG but remains unaltered after RYGB.^40^

Plasma leptin levels are decreased by all the three procedures; however, this is not followed by the compensatory changes in eating behaviour that accompany diet-induced weight loss. The increase in anorexigenic gut hormones seen after RYGB and VSG and the increased vagal stimulation after AGB appear to be enough to counterbalance the effects of lower leptin levels on hunger and weight regain. There is no evidence from animal models to suggest that leptin sensitivity is improved beyond that expected for weight loss *per se*.^29^

More recently, other novel mediators that may contribute to weight loss have surfaced. In a series of very elegant experiments, the transfer of gut microbiota from mice that have undergone RYGB to germ-free mice leads to 5% weight loss in the latter group, potentially through altered short-chain fatty acid production and signalling.^41^

### T2DM after bariatric surgery

#### Beta cell function

All bariatric surgery procedures cause glycaemic improvements through weight loss *per se*; however, improvements are more pronounced after RYGB and VSG and start becoming apparent before weight loss.^42, 43^ A potential reason underlying this may be the improvements in β-cell function after these procedures. After both RYGB and VSG, the early postprandial release of insulin is increased within a few days after surgery but also remains exaggerated after weight loss has been achieved.^44, 45, 46, 47^ On the contrary, total insulin release (expressed as area under the curve) is appropriately reduced after weight loss and in the face of reduced peripheral insulin resistance.^45^ AGB results in gradual weight loss and appropriate reductions in total postprandial insulin release but without the exaggerated early responses seen after RYGB or VSG.^45^

A human mechanistic study has shown that the early increases in insulin release after RYGB is partly because of the incretin effect of GLP-1.^48^ However, it has also been suggested that the negative energy balance achieved after bariatric surgery may be partly responsible for the early marked improvement of β-cell function.^49^. Even though this theory may well apply in the context of calorie intake reduction after bariatric surgery, it does not explain the very different insulin release profiles between RYGB/VSG and AGB.

#### Insulin resistance

The RYGB, AGB and VSG procedures facilitate reductions in peripheral insulin resistance directly as a result of gradual weight loss.^50^ Interestingly, however, in some human studies, hepatic insulin resistance, is reduced within days after RYGB before any significant weight loss has taken place.^51, 52^ Whereas this may be a direct result of a negative energy balance,^49^ the reduction in hepatic insulin resistance in some studies is greater in patients after RYGB compared with patients undergoing similar caloric restriction after AGB or a very-low-calorie diet.^44, 51, 52^ Even though these findings are not universal, they suggest that the bypass of the proximal bowel may have caloric intake and weight loss-independent effects on hepatic insulin resistance and hepatic glucose output.

#### Gut nutrient sensing

The role of portal vein glucose sensing became apparent in an elegant study of animal models that underwent a modified bypass procedure in which intestinal glucose production was increased after surgery. This led to higher levels of glucose in the portal vein and triggered a neural cascade through afferent and efferent vagal fibres resulting in lower hepatic glucose output.^53^ In animal models of the duodeno–jejunal bypass, jejunal nutrient sensing has also been shown to exert profound control on hepatic glucose output in a similar manner.^54^ Exposure of the jejunum to undigested nutrients following RYGB (and perhaps VSG) may explain the reductions in hepatic glucose output seen early surgery; however, such mechanistic studies have not been performed in humans as yet.

#### Bile acids

The search for the mechanisms underlying weight loss and glycaemic improvements after bariatric surgery is gradually revealing numerous novel metabolic targets. Whereas gut hormones affect insulin secretion after bariatric surgery, bile acids may also exert a significant physiological effect. Bile acids have been shown not only to increase gut hormone production but also to reduce food intake, gluconeogenesis, insulin resistance and even increase energy expenditure through their actions on the FXR and TGR-5 receptors in the periphery and/or in the brain.^55^ Plasma bile acids are elevated after RYGB and VSG, but not AGB, and negatively correlate with post-prandial glycaemic excursions.^56, 57^ However, no mechanistic studies have clearly defined their role after RYGB or VSG so far.

Table 1 summarises the available evidence on the physiological changes that take place after bariatric surgery.

### Lifestyle modification

Appetite reduction is a compelling goal for long-term weight loss and the resolution of the metabolic effects of morbid obesity. The appeal of abundant highly palatable food that influences eating patterns can make caloric restriction an unachievable goal for many individuals. In obese patients who have achieved weight loss, clinical data suggest the presence of compensatory mechanisms that may lead to weight regain.^58^ One study reported that overweight and obese patients who underwent a 10-week weight loss programme had significantly lower levels of leptin, PYY, CCK, insulin and amylin and significant increases in ghrelin levels from baseline. These differences persisted at 1 year and were accompanied by significant increases in appetite and preoccupation with food.^58^

Therefore, once weight loss has been achieved, appetite control is critical to prevent subsequent regain of weight. The effect of nutrient content on weight maintenance was examined in overweight or obese adults who had achieved at least an 8% reduction in body weight with a very-low-calorie diet.^59^ Individuals who completed a high-protein, low-glycaemic index diet regimen for 26 weeks following initial weight loss exhibited a higher maintenance rate of weight loss compared with those who completed a low-protein, high-glycaemic index diet. Furthermore, individuals assigned to the high-protein, low-glycaemic index diet continued to lose weight over the course of the study, whereas participants assigned to the low-protein, high-glycaemic index diet demonstrated significant weight regain. Diets high in protein and low in carbohydrates appear to be superior in the reduction of hunger and their ketogenic effects may have a role.^60^

In the context of T2DM, a very-low-calorie diet of 600 kCal had impressive short-term effects in reducing peripheral and hepatic insulin resistance, hepatic glucose output, and liver and pancreatic triacylglycerol.^49^ The diet also led to improvements in β-cell function and the authors concluded that part of the pathophysiology of T2DM can be reversed by energy intake restriction. This study sent a positive message to patients; however, the long-term success rate of such a strict dietary modification remains uncertain. However, even with little or no weight loss, high-protein, low-carbohydrate, low-glycaemic index and Mediterranean diets can also reduce cardiovascular risk or events in patients with or without T2DM.^61, 62^ More recently, the LOOK AHEAD RCT reported that intensive lifestyle modification, including only moderate caloric restriction, resulted in higher rates of T2DM remission at 4 years (7.3%) compared with standard education (2.0%).^63^ Even though the authors commented that these rates were low in absolute terms and did not translate in superior improvements in cardiovascular outcomes,^64^ the patient population was relatively old, had long T2DM duration and the criteria used for remission were stringent. These results suggest that early and intensive lifestyle intervention in T2DM can have positive effects and even obviate the need for medications in some patients for a reasonable length of time during the course of the disease.

### Obesity pharmacotherapy

The ability to achieve weight loss and maintenance with pharmacological agents has been an attractive yet elusive goal. For instance, although drugs such as rimonabant and sibutramine were previously used as a pharmacological strategy for obesity management, these agents were withdrawn following reports of increased risks of psychological and cardiovascular adverse events, respectively.^65, 66^ The discontinuation of these medications has not only led to longer and more thorough assessment of adverse events before and after drug marketing but also hopefully to the development of weight-loss agents that are more selective in their mode of action either in the brain or in the periphery.

Orlistat is the only drug that has stood the test of time in many countries around the world. It decreases fat absorption by 30% through the inhibition of pancreatic and gastric lipase. In practice, it works by making patients consciously reduce their fat intake to avoid unpleasant and socially embarrassing oily diarrhoea. A number of RCTs and meta-analyses have shown small decreases in weight of 2.9 kg,^67^ with beneficial effects on cardiometabolic risk factors and reductions in the incidence of T2DM.^68^ Gastrointestinal side effects remain common, whereas the rare occurrence of severe liver injury has also been reported.^67^

After 13 years with no new drug approvals, in 2012 the US FDA sanctioned two agents that reduce appetite and lead to modest weight loss. Lorcaserin is a serotonin 2C (5-hydroxytryptamine [5-HT]_2c_) agonist that leads to reductions in food intake and perhaps food reward. The 5-HT_2C_ receptor is not only located in the hypothalamus but also in some of the reward areas of the brain, including the prefrontal cortex, nucleus accumbens and amygdala. Phase 3 trials of 52-week duration have demonstrated that patients on the 10-mg, twice-daily dose lose an extra 2.9–3.6% of weight compared with patients on placebo.^69, 70, 71^ This was also accompanied by a 0.5% absolute decrease in HbA1c, improvements in lipids, blood pressure and waist circumference and no significant increase in the rate of cardiac valvular disease compared with placebo. The most frequent side effects of headache, dizziness, dry mouth and nausea were mild and tolerated by most patients, whereas symptomatic hypoglycaemia was reported significantly more frequently by diabetic patients in the intervention compared with the placebo arm.

The combination of phentermine and the antiepileptic topiramate has also been recently approved by the US FDA. Phentermine is a sympathomimetic drug, and its use with fenfluramine was discontinued in 1997 because of the association of fenfluramine with cardiac valvular disease and pulmonary arterial hypertension. Topiramate attenuates appetite through mechanisms that remain to be elucidated. The high-dose combination of the two medications led to an impressive 7.5–8.7% placebo-adjusted weight loss, a 0.4% absolute reduction in HbA1c and a decrease in the incidence of T2DM at 2 years compared with placebo.^72, 73, 74^ Side effects included paraesthesiae, constipation and dry mouth. Even though small increases in heart rate (by 1.7 beats per min, but no adverse clinical events) and discontinuation of the drug because of depression/anxiety and cognitive impairment were observed, the use of controlled release topiramate and lower doses of phentermine, as a fixed dose combination, may reduce the incidence of these potentially serious and problematic adverse events. The drug's longer-term effects on cardiovascular and psychiatric morbidity remain to be determined.

In summary, the two novel centrally acting medications may mimic in part the effect of bariatric surgery in reducing hunger; however, certainly their initial use should be cautious, and close long-term patient monitoring is essential.

### Modern T2DM pharmacotherapy

The drug discovery output for T2DM drugs has been much more fruitful and successful, mainly owing to the use of gut hormone analogues, which mimic endogenously produced ‘natural' signals, rather than entirely synthetic agents with potentially diffuse receptor affinity.

#### GLP-1 receptor agonists

Apart from its anorexigenic effects, GLP-1 acts as an incretin hormone to increase insulin release and lower glucagon secretion and hepatic glucose output.^75^ The four available GLP-1 receptor agonists are the short-acting exenatide twice-daily (BID) and lixisenatide, and the longer-acting exenatide extended release (ER) and liraglutide once daily. Head-to-head trials between exenatide BID and ER have shown the superior efficacy of the once-weekly preparation, which in addition to oral therapy, reduced HbA1c by 1.6–1.9% and weight by 2.3–3.7 kg compared with respective absolute reductions of 0.9–1.5% and 1.4–3.6 kg with exenatide BID.^76, 77^ Trials comparing liraglutide with exenatide BID in addition to oral therapy have shown that at a dose of 1.8 mg liraglutide was more effective in reducing HbA1c (absolute reduction 1.12% versus 0.79%) but not weight (−3.2 versus −2.8 kg; not significant).^78^ These medications reduce blood pressure in a weight-independent manner and even improve β-cell function as assessed by the homoeostasis model assessment of β-cell function and proinsulin/insulin ratio.^79, 80^ The superior efficacy of liraglutide may be because of its lower immunogenicity.^81^ Lixisenatide was the last GLP-1 receptor agonist to be launched in the market. On the basis of the data from the GetGoal programme of clinical trials, it leads to placebo-controlled HbA1c reductions of 0.3–0.9% (starting HbA1c ∼8.0–8.5%) and weight changes of −2.7 to +0.3 kg (starting body mass index ∼30–34 kg m^−2^).^82, 83^ Lixisenatide is particularly effective in controlling postprandial glucose excursions and may therefore be clinically useful when used in combination treatment with long-acting insulins. Whereas these compounds are currently used in the management of T2DM, an expanded role of high-dose liraglutide in weight-loss initiation and maintenance is currently under investigation.^84^

A major advantage of these drugs is their relatively favourable side effect profile. On the basis of the trials mentioned previously, the rates of hypoglycaemia are relatively low, whereas nausea, vomiting and diarrhoea, although common, are well tolerated by most patients. Nausea tends to be transient in the majority of cases and is less common with the longer-acting preparations.^84^ Longer-term studies are necessary to determine cardiovascular and safety outcomes, and in particular, answer specific concerns over the association with pancreatitis, pancreatic and thyroid C-cell cancer, and increases in heart rate with exenatide and liraglutide.

#### DPP-4 inhibitors

The four available dipeptidyl peptidase-4 (DPP-4) inhibitors are sitagliptin, vildagliptin, saxagliptin and linagliptin. They all inhibit the enzyme that degrades GLP-1, thus raising the tissue levels. The major advantage of these medications is their oral route of administration and the relatively low incidence of hypoglycaemia and gastrointestinal side effects. Compared with GLP-1 agonists, they are less effective in improving glycaemia (HbA1c absolute reductions of 0.5–1.0%) and are weight neutral.^85^ It is interesting to note that DPP-4 inhibitors are only slightly less efficacious compared with GLP-1 agonists, even though plasma levels while on DPP-4 treatment are significantly lower to those seen when on GLP-1 analogue therapies. A number of elegant studies have shown that the majority of physiological action of DPP-4 inhibitors is exerted through local, and not just plasma, increases in GLP-1 and stimulation of the gut wall and portal vein vagal receptors.^86, 87^ As DPP-4 is also necessary for the activation of anorexigenic gut hormones, such as PYY, its inhibition may be responsible for the weight neutrality of these agents.

#### Amylin mimetics

Pramlintide is an injectable amylin-mimetic that has been approved by the FDA for the treatment of T1DM and T2DM. Amylin is a hormone co-secreted with insulin by the β cell and has multiple physiological actions including the reduction in gastric emptying, food intake, post-prandial glucose and glucagon release. In the context of T2DM, it has been shown to cause small placebo-subtracted absolute reductions in HbA1c of 0.2–0.4% (even with high starting HbA1c of 8.2–9.3%) and weight loss of 2.1–2.3 kg (starting weight 97–103 kg).^88^ Side effects include temporary nausea and mild to moderate hypoglycaemia in T2DM. Its high cost and injectable mode of delivery have somewhat limited its clinical use.

#### SGLT-2 inhibitors

The newest class of agents released for clinical use in some parts of the world is the sodium-glucose co-transporter 2 inhibitors that lower plasma glucose, independently of insulin resistance and secretion, by reducing renal reabsorption.^89^ The placebo-subtracted absolute reductions in HbA1c with the first agent of this class, dapagliflozin, are modest (0.4–0.8%, starting HbA1c ∼7.8–8.0%), but are maintained at least up to 2 years, whereas the excretion of glucose is beneficial for weight loss (2.0–3.0 kg).^89^ Dapagliflozin also leads to reductions in systolic blood pressure of 4–5 and diastolic blood pressure of 2–3 mm Hg, partially as a result of its diuretic effects and the inhibition of the renin–angiotensin system in the kidney. The route of administration of dapagliflozin is oral and the rates of hypoglycaemia with the drug are low. The major benefit of this class of drugs is that they can be used at any stage of the disease process, in combination with most other glucose-lowering medications, and may even preserve beta cell function. Adverse effects include urinary tract and genital infections and volume depletion, and there are concerns regarding the rare incidence of breast and bladder cancer and liver injury.^89^

In summary, the treatment of T2DM has undergone a metamorphosis in the last 7 years or so because of the addition of a number of new classes of glucose-lowering medications. These not only have reasonable effects on glycaemia but, more importantly, promote clinically significant weight loss. However, long-term data on cardiovascular outcomes and serious adverse events are vital before these agents' position in the treatment algorithm for T2DM is secure.

### Devices

The most promising of the devices currently in use as less invasive treatments for obesity and T2DM is the duodenal–jejunal bypass liner (EndoBarrier, GI Dynamics, Lexington, MA, USA). It is made of a plastic polymer, is 60 cm in length and is inserted endoscopically in such a way as to line the proximal small bowel and exclude contact of food with its walls. By mimicking the physiological changes in the biliopancreatic limb (one of the components of RYGB), this device leads to weight reductions of 10–20%.^90, 91^ Its metabolic effects are more impressive, with absolute reductions in HbA1c of ∼1.2–2.3% (starting HbA1c 7.3–9.1%).^92, 93, 94^ The device lowers fasting glucose within 1 week of insertion; however, the mechanism of action remains unclear. The early glycaemic improvements suggest beneficial effects on hepatic insulin resistance and hepatic glucose output. The exposure of the proximal small bowel to undiluted bile acids and of the distal small bowel to undigested nutrients may have a vital physiological role in the efficacy of this device.^54^ The major limitations of this intervention are that it has to be explanted electively at 6–12 months and may need to be prematurely removed because of severe abdominal pain, vomiting, obstruction and bleeding in up to 30% of cases. As with all medical devices, it needs to undergo further comprehensive evaluation through clinical trials before it is widely used in clinical practice.

Gastric stimulators, which are inserted endoscopically, work by reducing hunger and increasing satiation. Their mode of action is unclear but may include inhibition of efferent vagal signalling resulting in gastric detention, reduced gastric emptying and therefore early satiation.^95^ They may also have stimulatory effects on afferent vagal fibres, which signal satiation to the hypothalamus via the brainstem. Even though open-labelled studies reported promising results, the findings from RCTs have been less encouraging.^96^ Nevertheless, there is room for the technology to improve and promote weight loss more safely than bariatric surgery.

Gastric balloons are fluid-filled silicone sacs inserted endoscopically and reduce weight by increasing early satiety. The current published studies have not shown their superiority to conventional weight-loss treatments.^97^ However, gastric balloons can still be useful as a temporary measure to reduce anaesthetic risk and therefore act as a bridge to elective orthopaedic, bariatric, cardiac or transplantation surgery.

## Discussion and Conclusion

So can we mimic the clinical efficacy of surgery with less invasive therapies? On the basis of the most recent studies, the answer is that at the moment we cannot, but we are getting closer. The SOS study compared bariatric surgery to lifestyle and behavioural interventions. The study started recruiting patients in 1987, and modern obesity pharmacotherapy was not approved in Sweden until 1998. Additionally, the lifestyle support that patients received was very heterogeneous and in some cases not intensive at all. Nevertheless, weight loss and glycaemic improvement outcomes were far better with surgery, even after 20 years of follow-up.^3, 4, 98, 99^

The most recent RCTs, which have focused on patients with T2DM, tell a slightly different story. In a head-to-head trial between biliopancreatic diversion, RYGB and modern medical care, the surgical groups did better in terms of glycaemic control and weight loss than the medically treated group, but to a lesser degree when compared to the SOS study.^5^ Medical patients lost 4.7% of weight, their HbA1c improved by 8.4% (relative reduction) and the blood pressure and lipid outcomes were not statistically different compared with the RYGB group at 24 months. In an RCT that compared RYGB with VSG and modern medical care, the patients in the latter group also lost weight, improved their glycaemic control, cardiometabolic factors and inflammation at 12 months.^6^ Whereas surgery was still superior to medical care, the combination of the two was synergistic in its positive outcomes. Even though the combination of surgical and non-surgical interventions has been practiced successfully for years in other fields of metabolic and endocrine medicines (for example, pituitary disease), the concept of providing bariatric surgery as part of a treatment algorithm, together with—and not instead of—less invasive therapies is novel and one that is increasingly popular.^6, 8^

The most up-to-date lifestyle modification therapies, pharmacotherapy and medical devices were only partially studied in these trials. A ‘medical bypass' alternative to bariatric surgery may not be as far in the future as we previously thought. Weight loss of significant magnitude could potentially be achieved through the use of lorcaserin, phentermine/topiramate, orlistat, gut hormone analogues and medical devices, not in isolation but in combination. In the context of T2DM associated with obesity, we would suggest that the effect of RYGB on reducing hepatic insulin resistance can be mimicked by the endoscopically placed duodenal–jejunal bypass liner and that some of the effect of RYGB on enhanced insulin secretion can be mimicked by GLP-1 agonists. In addition, there may be a synergism to reduce body weight between the endoscopically placed duodenal–jejunal bypass liner, GLP-1 agonists, DPP-4 inhibitors, pramlintide and SGLT-2 inhibitors. The weight loss and glycaemic improvements induced by these less invasive treatments could then be maintained on a high-protein, low-glycaemic index or Mediterranean diet. The clinical efficacy and safety of such combination therapies, together with gut hormone analogues and bile acid receptor agonists currently under development, could be compared with that of bariatric surgery in large-scale RCTs. These trials will inform us as to whether the beneficial clinical and physiological effects of bariatric surgery can be mimicked but also whether this can be achieved with a reduced morbidity and mortality burden.

We conclude that the physician's armamentarium against obesity and associated T2DM is getting stronger through the use of specific dietary modifications, novel medical devices and pharmacotherapy. Although there are no magic bullets, an integrated multimodal approach may yield success. Non-surgical interventions that mimic the metabolic benefits of bariatric surgery remain tenable alternatives for many patients and health-care professionals.

## Figures and Tables

**Table 1:** Physiological changes after the most commonly performed bariatric surgery procedures and modern obesity and type 2 diabetes mellitus pharmacotherapy

	*RYGB*	*VSG*	*AGB*	*Orlistat*	*Lorcaserin*	*Phentermine/topiramate*	*GLP-1 agonists*	*DPP-4 inhibitors*	*SGLT-2 inhibitors*	*Pramlintide*
Appetite	↓	↓	↓	↔/↑	↓	↓	↓	↔	?	↓
Plasma ghrelin	↑/↓/↔	↓	↑	↔/↑	?	?	↓	↔	?	↔
Plasma GLP-1	↑	↑	↔	↔/↑	?	?	↑	↑	?	↓
Plasma PYY	↑	↑	↔	↔/↓	?	?	↓ fasting levels	↓ PYY_3–36_/↔	?	↔/↓
Plasma Oxyntomodulin	↑	?	?	?	?	?	?	?	?	?
Plasma CCK	↔	↔/↑	?	↔/↓	?	?	?	?	?	↔/↓
Plasma leptin	↓	↓	↓	↓	?	?	↓	?	?	↓
Gastric emptying	↑/↓	↑	↔	↑	?	?	↓	↔	?	↓
Caloric malabsorption	Minimal for fat only	?	?	↑	?	?	?	?	?	?
Energy expenditure	↑/↓/↔	↔	?	↓	↓	?	?	?	?	↑/↔
Food preferences	↓ Consumption of fat and sugar	↓ Consumption of fat and sugar	↔ Or ↑consumption of fat and sugar	↓ Consumption of fat necessary	?	?	↓ consumption of fat and sugar	?	?	↔/↓consumption of fat
Glycaemic improvements	Early and sustained, weight-dependent and -independent	Early and sustained, weight-dependent and -independent	Gradual and sustained, weight-dependent	Gradual	Gradual	Gradual	Early and gradual alongside weight loss	Early and sustained	Early and sustained	Early and gradual alongside weight loss
Early postprandial insulin release	↑, Early and sustained	↑, Early and sustained	↔	↑, Gradual	?	?	↑	↔/↑	?	↓
Insulin resistance	↓	↓	↓	↓	↓	↓	↓	↔	↓	↓
Plasma bile acids	↑	↑	↔	?	?	?	?	?	?	?
Gut microbiota	Significant changes	?	?	?	?	?	?	?	?	?

Abbreviations: AGB, adjustable gastric banding; CCK, cholecystokinin; DPP, dipeptidyl peptidase; GLP-1, glucagon-like peptide-1; PYY, peptide YY; RYGB, Roux-en-Y gastric bypass; SGLT, sodium-glucose co-transporter; VSG, vertical sleeve gastrectomy; ↑, increase in parameter; ↓, decrease; ↔, no change; ?, not known.

Evidence has been provided predominantly by human and, when that was not available, animal studies.
